# Sustained Release of Antibacterial Agents from Doped Halloysite Nanotubes

**DOI:** 10.3390/bioengineering3010001

**Published:** 2015-12-23

**Authors:** Shraddha Patel, Uday Jammalamadaka, Lin Sun, Karthik Tappa, David K. Mills

**Affiliations:** 1 Center for Biomedical Engineering and Rehabilitation Science, Louisiana Tech University, Ruston, LA 71272, USA; nenomole@gmail.com (S.P.); uja002@latech.edu (U.J.); lsu002@latech.edu (L.S.); kkt007@latech.edu (K.T.); 2 Wayne State University, St. John Hospital & Medical Center, 22101 Moross Rd, Detroit, MI 48236, USA; 3 School of Biological Sciences, Louisiana Tech University, Ruston, LA 1272, USA

**Keywords:** antiseptics, antibiotics, drug release, halloysite nanotubes, nanocontainers, poly-*e*-caprolactone, nanocomposite mats

## Abstract

The use of nanomaterials for improving drug delivery methods has been shown to be advantageous technically and viable economically. This study employed the use of halloysite nanotubes (HNTs) as nanocontainers, as well as enhancers of structural integrity in electrospun poly-*e*-caprolactone (PCL) scaffolds. HNTs were loaded with amoxicillin, Brilliant Green, chlorhexidine, doxycycline, gentamicin sulfate, iodine, and potassium calvulanate and release profiles assessed. Selected doped halloysite nanotubes (containing either Brilliant Green, amoxicillin and potassium calvulanate) were then mixed with poly-*e*-caprolactone (PLC) using the electrospinning method and woven into random and oriented-fibered nanocomposite mats. The rate of drug release from HNTs, HNTs/PCL nanocomposites, and their effect on inhibiting bacterial growth was investigated. Release profiles from nanocomposite mats showed a pattern of sustained release for all bacterial agents. Nanocomposites were able to inhibit bacterial growth for up to one-month with only a slight decrease in bacterial growth inhibition. We propose that halloysite doped nanotubes have the potential for use in a variety of medical applications including sutures and surgical dressings, without compromising material properties.

## 1. Introduction

Among other natural materials studied for use in drug delivery, nanocoatings, and tissue-engineered scaffolds, halloysite has only recently been discovered. Halloysite nanotubes (HNTs) are naturally occurring clay nanoparticles found deposited in soils worldwide [1,2,3]. Halloysite is an economically viable raw material that can be mined from a deposit as a raw mineral and refined on location. Halloysite is structured as a two-layered aluminosilicate, chemically similar to kaolin and has a predominantly hollow nanotubular structure in the submicron range [2,3,4,5]. HNTs typically display an inner diameter ranging from 15 to 50 nm, an outer diameter from 30 to 50 nm, and a length between 100 to 2000 nm [1,2].

Halloysite nanotubes have high capillary forces; so they quickly adsorb numerous materials and within a wide range of pH. It has a negative electrical zeta-potential of *ca.* −50 mV, which imparts to HNTs suitable dispersibility in water-based polymers and other media. HNTs present a large outer surface area that may be functionalized and an inner lumen that can be loaded with different drugs or bioactive factors for sustained drug release [1,3,6]. This increases the drug effectiveness, without increasing concentration, as the drug is released slowly from the HNT lumen [1,6,7]. Drugs of smaller molecular size are typically vacuum-loaded within the inner lumen of the nanotube and drugs larger in molecular size can attach to the outer surface of halloysite. The inner lumen and outer surface are oppositely charged, and charge intensity is easily modified.

Halloysite nanotubes have been shown to be cytocompatible in studies using different cell types [2,4]. Human dermal fibroblasts cultured on HNT nanofilms showed no cytotoxic effects, proliferated and expressed tissue-specific proteins showing that they maintained their cellular phenotype on an HNT layered nanoparticle thin film [8]. Mesenchymal stem cells also thrived on HNT nanofilms [9] and a recent study has provided support for HNT biocompatibility [10]. These properties support the concept of the use of HNTs as a nanocontainer and nanocarrier able to entrap biologically active agents within the inner lumen, followed by their retention, and slow release [11]. Ruling *et al.* [12] and Patel [13] established the concept of using doped HNTs and the electrospinning technique to fashion drug doped polymer fiber composites (poly(lactic-co-glycolic acid) and polycaprolactone, respectively) for sustained release. Several recent studies have extended this work and fabricated electrospun HNTs/polymer composites for delivery of a diverse set of drugs. Xue *et al.* [14] doped metronidazole into HNT poly(caprolactone)/gelatin microfibers and showed that such a nanocomposite could extend drug release to 20 days *versus* only six days from plain microfibers [14]. Polylactic acid and halloysite composites have seen extensive study in conjunction with halloysite for applications ranging from drug delivery to tissue engineering [15,16]. Sun *et al.* [17] have taken this work further by showing the HNTs can be encapsulated in a drug-infused polyelectrolyte coating and doped into nylon-6. The composites were electrospun into fiber mats and sutures and reduced osteosarcoma cell proliferation *in vitro* [17]. Sharma *et al.* [14] offers an excellent review of nanofibers and their biomedical applications [18].

The current study demonstrates that a wide range of antibacterial agents can be loaded into the HNT lumen and slowly released. Furthermore, HNTs doped with chlorhexidine, povidone iodine, Brilliant Green were incorporated into PCL nanocomposite mats. These mats, when applied to confluent bacterial cultures, maintained an anti-bacterial growth inhibition field for up to one month. We propose that HNT doped-mats containing single or mixed sets of antibiotics/antifungals can be used for maintaining a sterile field in body cavities and on body surfaces or used to control or eliminate bacterial growth in contaminated wounds. Such scaffolds could also be loaded with growth factors (and other drugs) forming a mixed nanocomposite that would enhance healing leading to speedier wound repair and patient recovery.

## 2. Experimental Section

### Materials

Halloysite nanotubes were purchased from Sigma Aldrich (St. Louis, MO, USA). The outside diameters of the halloysite vary from 50 to 70 nm, the average inner diameter is 15 nm and their lengths varies from 1 to 1.5 µm [6]. Brilliant Green is a topical antiseptic commonly used in Eastern Europe and Russia and is used to treat various skin and mucosal infections as well as sinus infections. Chlorhexidine is a chemical antiseptic that is effective on both Gram-positive and Gram-negative bacterial and is commonly used to treat skin infections, as a skin wash, and in mouthwash as a treatment for gingivitis. Iodine and povidone-iodine are commonly used antiseptics with povidone-iodine the universally preferred iodine antiseptic. Brilliant Green, chlorhexidine, iodine, and polycaprolactone were obtained from Sigma Aldrich (St. Louis, MO, USA) and povidone-iodine from Wal-Mart, Bentonville, AR. Amoxicillin and potassium calvulanate are both antibiotics grouped in a class of drugs called penicillins and were purchased from Macleods Pharmaceuticals, India. Doxycycline is a tetracycline antibiotic used to slow bacterial growth and was obtained from Shreya Life Science PVT. LTD. Roorkee, India. Nitrofurantoin is an antibiotic usually used in the treatment of urinary tract infections (Sigma Aldrich, St. Louis, MO, USA).

## 3. Methods

### 3.1. Halloysite Drug Loading

To study the release pattern of drugs from HNTs, chlorhexidine, povidone iodine, Brilliant Green, iodine, doxycyclin, amoxicillin and potassium calvulanate were loaded into halloysite nanotubes. For this set of experiments, 20 mg of each drug was dissolved in 1 mL of water or alcohol, and the mixture was sonicated until the drug was dissolved. Once the solution became transparent, 50 mg of halloysite powder was added. The mixture was sonicated again for 30 min. Then, the solution was placed in a vacuum chamber, and a vacuum applied for 20 min. When the vacuum is applied, air bubbles are removed from the halloysite and removal of the vacuum causes the drug solution to enter the halloysite lumen. After 20 min, the vacuum was stopped, and the tube containing doped halloysite was removed from the vacuum chamber and kept at room atmosphere for 20 min. This vacuum process was repeated three times, followed by washing with water to remove any unloaded drugs. As povidone-iodine comes prepared in solution form, HNTs (50 mg) were directly added into a 1 mL solution of povidone-iodine and loaded as described above. For all experiments, six samples per time or testing point were used. All experiments were repeated twice and results from both sets of experiments were in accord.

### 3.2. Fabrication of Drug Loaded Halloysite-PCL Scaffolds

#### 3.2.1. Preparation of PCL Solution

A 9-wt% PCL-chloroform mixture was used for the producing electrospun PCL scaffolds. The PCL solution was prepared by sonicating PCL beads in chloroform. PCL beads were dropped one by one until a viscous homogenous PCL solution was formed. This took roughly 1.5 h and as chloroform evaporates quickly, additional chloroform was added to maintain volume.

#### 3.2.2. Preparation of PCL and HNT Solution

For PCL mats, a 5 and 7-wt/% ratio of HNTs to PCL scaffold solution was used. It was prepared by the addition of 5 or 7-wt/% HNTs or antibacterial-doped HNTs (Brilliant Green, amoxicillin and potassium calvulanate) into the PCL-chloroform mixture. The PCL/chloroform/HNT solution was sonicated for 10 min, followed by electrospinning. Several variations on this method were also tested.

#### 3.2.3. Electrospinning HNT/PCL Mats

The method of electrospinning was used to prepare antibacterial drug loaded HNT/PCL mats. The electrospinning set up consisted of a syringe pump, syringe, a collector plate, and a high voltage electricity source. The entire electrospinning set-up was placed in a Plexiglas housing to enclose and stabilize the path of the polymer jet.

**Figure 1:**
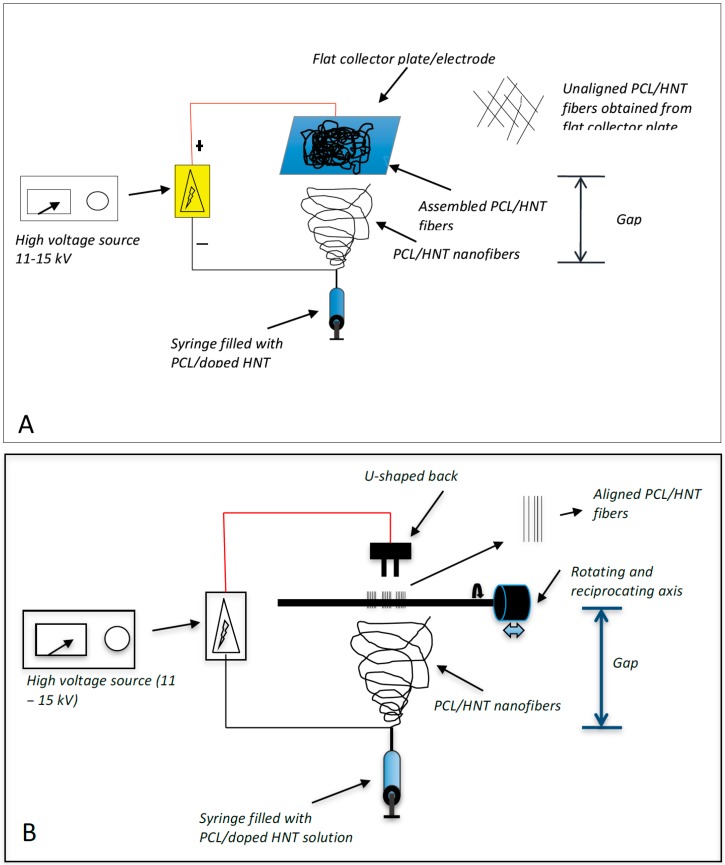
Electrospinning assembly set-up. (**A**) shows the assembly system that produced woven PCL fibers with no fiber orientation; (**B**) shows the assembly system that produced oriented (uniaxial) PCL fibers.

Two different electrospinning configurations were used (Figure 1). For the fabrication of woven HNT/PCL fibers, 1 mL of the PCL/chloroform/HNT mixture was loaded into a 1 mL syringe. A syringe pump was used to apply constant pressure (1 mL/hr.) to the syringe and the PCL/chloroform/HNT solution released at 10 µL/minute using flat head needles to dispense the polymer solution. A high voltage (17–20 kV) was maintained between the tip of the syringe needle and the collector plate. A polymer jet is then formed, the solvent evaporates, and the loaded HNT/PCL mats were assembled on the surface of an aluminum collector plate (Figure 1A). The distance between the tip of the needle and the collector plate was maintained at 20 cm for assembly of the antibacterial drug loaded mats.

For scaffolds with an oriented HNT/PCL fiber arrangement, an electrospinning set-up similar to that for non-woven scaffolds was employed with the exception of the collecting apparatus (Figure 1B). For oriented fibrous scaffolds, a “U”-shaped collector, located 1 cm from the collector, was connected to the ground. The distance between the tip of the needle and the collector plate was maintained at 17 cm for assembly of the antibacterial drug loaded halloysite-PCL scaffolds. Briefly, 1 mL of the PCL/chloroform/HNT mixture was loaded into a 1 mL syringe and the pump was used to apply constant pressure (1 mL/hr.) to the syringe and the PCL/chloroform/HNT solution released at 10 µL/minute using flat head needles to dispense the polymer solution. Loaded PCL/HNT fiber nanocomposites were assembled as oriented fibers onto a rotating drum instead of the collector plate.

Amoxicillin, Brilliant Green and potassium calvulanate were successfully loaded in halloysite-Poly-lactic acid (PLA) and halloysite-polyethylene oxide (PEO) mats. However, these mats showed low tensile strength and were rejected for further testing (data not shown).

### 3.3. Drug Release from Drug Loaded Halloysite Nanotubes

Drug loaded HNTs were added to 1 mL of water and placed on a magnetic stirrer for 10 min. The tubes containing the solution were then centrifuged at 7000 rpm for 2 min. The supernatant was removed and 1 mL of water was added to the tube, and resuspended. The tube was again incubated on the magnetic stirrer for 10 min, followed by centrifugation at 7000 rpm for 2 min. This process was repeated at selected time intervals. Magnetic stirrer was removed and the amount of antibacterial drug released was measured with a UV Spectrometer (Cole Parmer, Vernon Hills, IL, USA). For all drug release studies, the assays performed were repeated twice.

### 3.4. Bacterial Studies

We used confluent cultures of *E. coli* and *S. aureus* to test the efficacy of HNT/PCL mats. In these bacterial experiments, 1 L of LB broth was prepared by mixing 10 grams of NaCl, 10 grams of tryptone, 5 grams of yeast extract, 15 grams of agar, and enough distilled water to make a final volume of 1 L. This LB broth was sterilized by autoclaving at 121 °C for 15 min. After autoclaving, LB broth was poured into Petri dishes, which were allowed to solidify over night. The next day, a loop of *E*-*coli* and *S. aureus* bacteria was spread on the LB broth plates. Bacteria were allowed to grow until confluent. At confluence, HNT/PCL nanocomposites loaded with *amoxicillin*, Brilliant Green, chlorhexidine, and provodine iodine, were applied to the surface of bacterial cultures. Serial cultures of bacteria were used to assess the duration of release from HNT doped scaffolds. HNT doped scaffolds were removed from spent bacterial cultures and applied to a fresh confluent bacterial culture, observed and photographed and the process repeated for over a one-month period.

## 4. Results

### 4.1. Morphology

**Figure 2:**
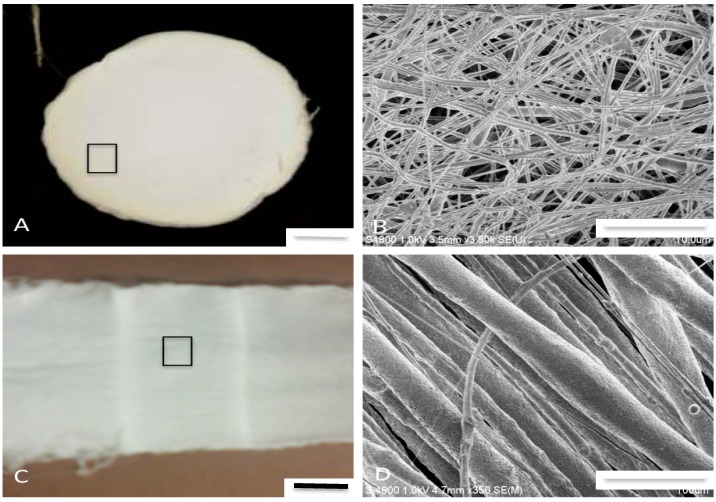
Fabricated PLC/Halloysite nanocomposites with 5% (**A**,**B**) and 7.5% HNTs (**C**,**D**). (**A**) Photograph of 5% HNT/PCL nanocomposite with a random fiber pattern. Area outlined by square shown under SEM microscopy in (**B**); (**B**) SEM image of 5% HNT/PCL nanocomposite, insert a single fiber at higher power; (**C**) 7.5% HNT/PCL nanocomposite with an oriented fiber pattern. Area outlined by square is shown at higher magnification in (**D**); (**D**) SEM image of 7.5% HNT/PCL nanocomposite, containing 5% HNTs in electrospun PCL. Note the PCL fibers have a defined orientation. Scale bars = 2 centimeters in (**A**,**C**), 10 microns in (**B**) and 100 microns in (**D**).

Our results suggest that the speed of the target and the form of the collector plate dictates the degree of fiber anisotropy and diameter within the forming HNT/PCL scaffold. The addition of HNTs up to 15% did not significantly affect the fiber pattern or porosity (data not shown). With modest changes in target speed and collector design, scaffolds could be produced with a range of desired alignments, from random patterns with large pore size (Figure 2A,B) or at the slower speeds a more oriented fiber pattern (Figure 2C,D). Figure 2B,D shows clear differences in fiber diameter and porosity.

### 4.2. Contact Angle Measurements

**Figure 3:**
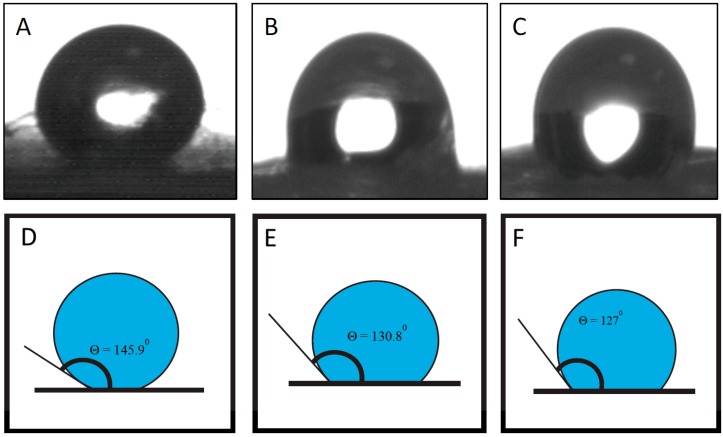
Contact angle for random-oriented PCL mats and PCL mats loaded with HNTs. (**A**) Pure PCL (145.9°); (**B**) PCL with 5% HNT (130.8°); and (**C**) PCL with 10% HNT (127°). *n* = 6. (**D**–**F**) graphically illustrated contact angles shown in (**A**–**C**).

The contact angle measurements of PCL and PCL/HNT nanocomposite scaffolds were performed. Pure PCL is hydrophobic in nature (Figure 3A). Results of contact angle measured showed that PCL/HNT nanocomposite scaffolds showed increased hydrophilicity with increased HNT content (*i.e.*, decreased the contact angle, Figure 3B,C). Decrease in contact angle implies more wetting of the surface. Wetting of the mats results in better water flux into the scaffold leading to better drug elution. Moreover, cell adhesion is favored on hydrophilic surfaces.

### 4.3. Antibacterial Drug Release from Drug-Loaded Halloysite

**Figure 4:**
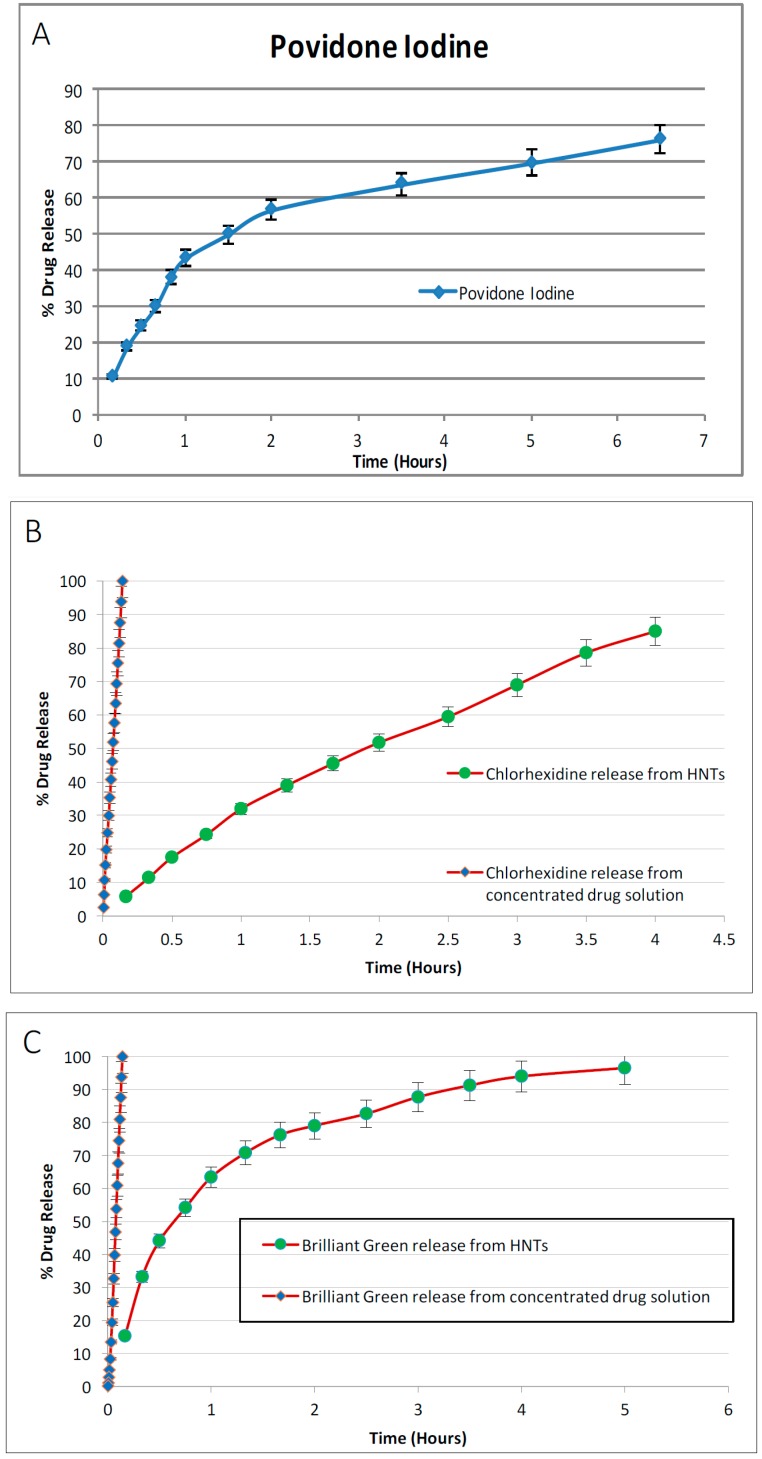

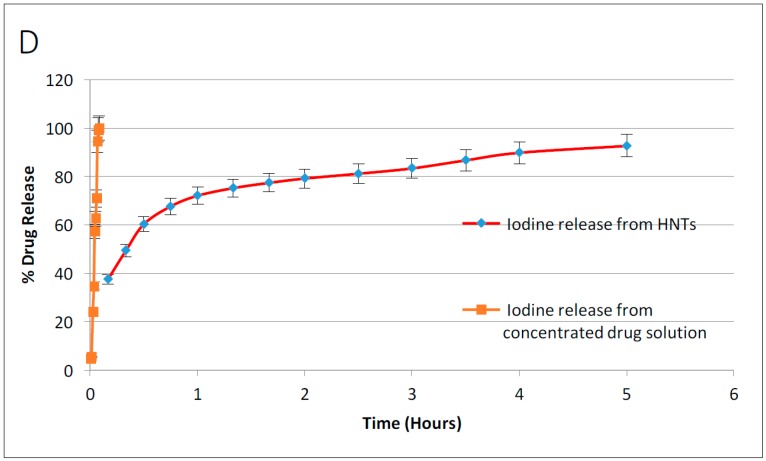
Release profile of antiseptics from HNTs. (**A**) Povidone iodine; (**B**) Chlorhexidine; (**C**) Brilliant green; (**D**) Iodine. *n* = 4 trials.

**Figure 5:**
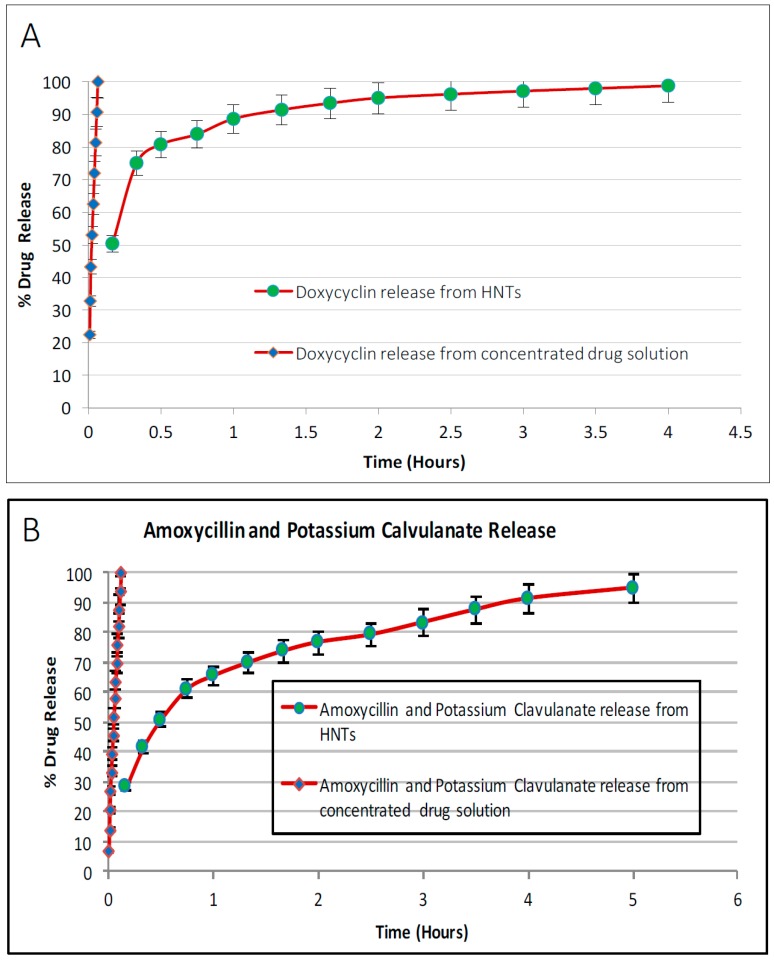
Antibiotic release from HNTs. (**A**) Doxycyclin; (**B**) Amoxycillin and Potassium Calvulanate. *n* = 4 trials.

**Figure 6:**
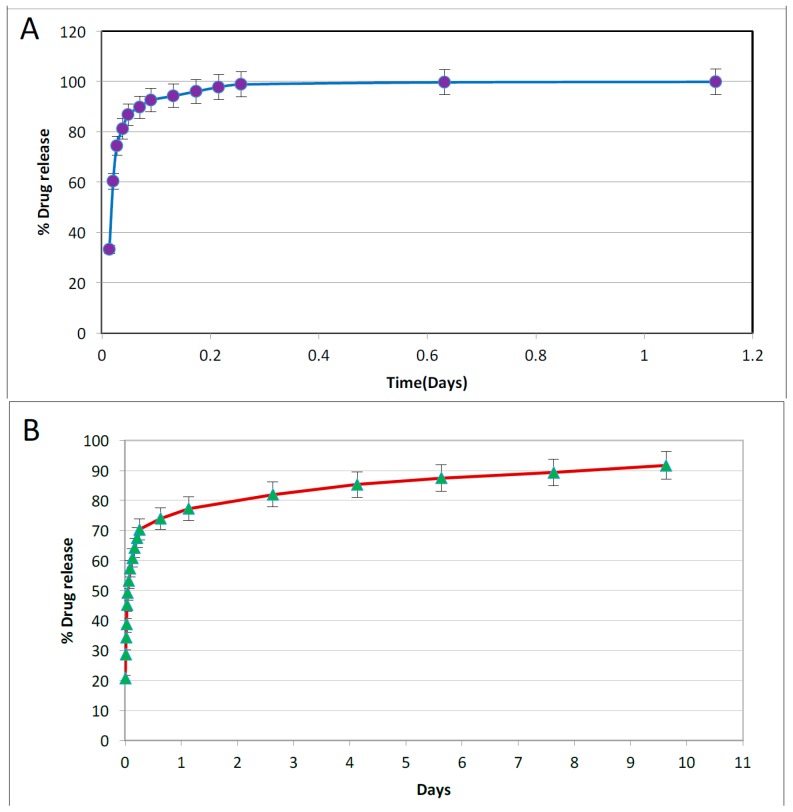
(**A**) Brilliant Green release from loaded halloysite-PCL scaffolds; (**B**) Amoxycillin and potassium calvulanate release from loaded halloysite-PCL scaffolds. *n* = 3 trials.

The drug release profiles for povidone iodine, chlorhexidine, Brilliant Green, iodine, doxycyclin, amoxicillin and potassium calvulanate are shown in Figure 4, Figure 5 and Figure 6. The concentration of the drug released from HNTs was measured by a UV spectrophotometer. As shown in Figure 4, 76% of povidone iodine from HNTs was released from HNTs in 6.5 h, 84.95% of chlorhexidine was released in 4 h, 96.52% of Brilliant Green was released in 5 h, and 92.68% of Iodine in 5 h. In Figure 5 release profiles of antibiotics are shown. 98.73% of doxycycline was released from HNTs in 4 h and HNTs loaded with both amoxicillin and potassium calvulanate showed a release rate of 94.83% over a 5-hour period. In almost all cases, despite extensive washing after loading, there was an initial burst of drug followed by a more gradual release pattern.

### 4.4. Antibacterial Drug Release from Halloysite-PCL Scaffold

Figure 6 shows drug release profiles from the Brilliant Green loaded halloysite-PCL scaffold and amoxicillin and potassium calvulanate loaded HNT/PCL scaffold, respectively. As shown in Figure 6, 99.95% of Brilliant Green was released from Brilliant Green loaded halloysite-PCL scaffold in 1.1 days. A sum total of 91.67% of *amoxicillin* and potassium calvulanate was released after 9.6 days from amoxicillin and potassium calvulanate loaded halloysite-PCL scaffold. Despite repeated rinses of doped scaffolds after loading, approximately, 15%–20% of the initial drug burst is probably associated with externally adsorbed dyes and antibiotics (Figure 6).

**Figure 7:**
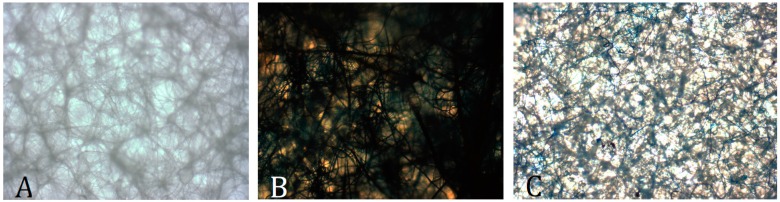
Fabricated PLC/Halloysite nanocomposite scaffolds loaded (and releasing) amoxicillin (**A**) Brilliant Green; (**B**) and Brilliant Green and amoxicillin (**C**). Photographed after one week. Brightfield microscopy, 4X.

Figure 7 provides visual evidence of doped PCL/HNT scaffolds and drugs released (amoxicillin, Brilliant Green and amoxicillin) from loaded HNTs. Even after one month, antiseptics and antibiotics were still being released from PCL scaffolds (data not shown).

**Figure 8:**
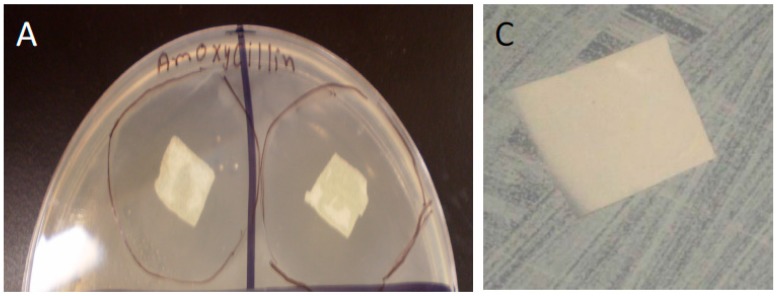

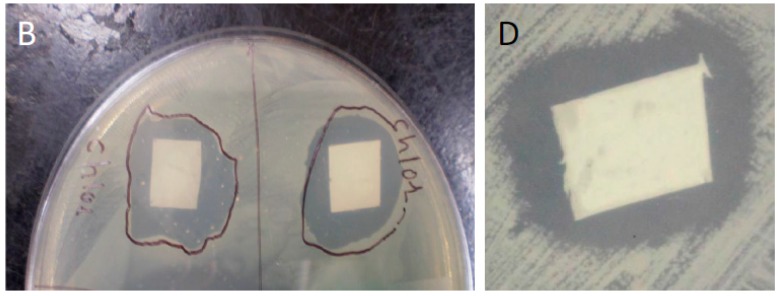
Doped HNT/PCL mats applied to confluent bacterial cultures. 5% HNT/PCL nanocomposites loaded with amoxicillin (**A**) and chlorhexidine (**B**) and applied to confluent cultures of *E. coli.* Black lines outline extent of anti-bacterial growth zone; (**C**) Zone of inhibition of *S. aureus* growth around empty halloysite-PCL scaffolds showing no antibacterial effect; (**D**) PCL mats with HNTs doped with Brilliant Green. (**A**–**D**) were photographed one week after application of doped mats.

**Figure 9:**
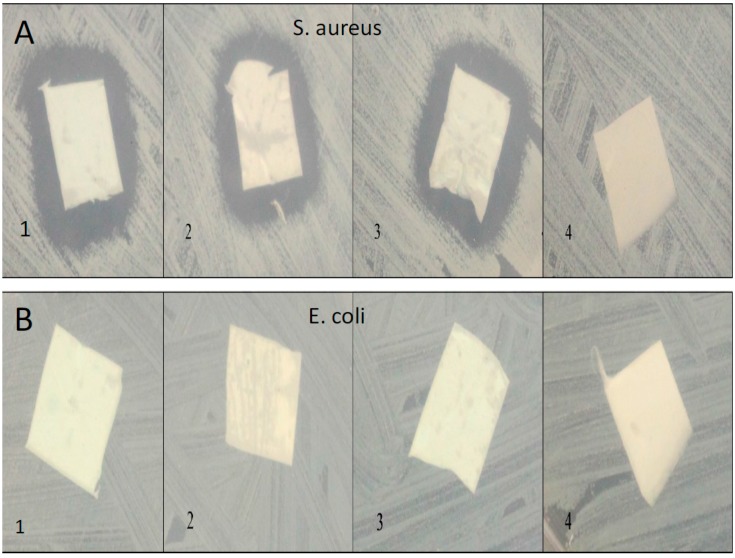
(**A**) *S. aureus* and (**B**) *E. coli* confluent cultures with scaffolds added and observed after 7 days. PCL mats prepared by combining the PCL solution and 7% HNTs before electrospinning, (**A1/B1**); PCL with 7% HNTs doped with Brilliant Green prepared by dispersing the HNTs in chloroform and then dissolving PCL within the solution using sonication, (**A2,3/B2,B3**); (**A4/B4**) PCL mats containing no Brilliant green or HNTs. Growth inhibition was observed in Brilliant Green doped HNT/PCL scaffolds and only on *S. aureus*. No observable difference between the two fabrication methods was observed.

### 4.5. Anti-Bacterial Effects of Doped HNT/PLC Nanocomposites

Confluent cultures of the bacteria, *E. coli* and *S. aureus*, were established and the anti-bacterial effects of doped HNT/PLC nanocomposites were assessed. Amoxicillin, Brilliant Green, chlorhexidine and povidone iodine were loaded into HNT/PCL scaffolds and studied for their ability to inhibit bacterial growth. Doped nanocomposites were effective or ineffective in creating zones of growth inhibition depending on the antibacterial applied and the bacterial strain. Zones of inhibition were apparent within hours after application and amoxicillin and chlorhexidine doped HNT/PCL mats remained effective on *S. aureus* up to one week (Figure 8). Variation in method of doped HNT/PCL solutions prior to electrospinning did not alter their effectiveness (inhibition zone size and duration of inhibition varied) (Figure 9).

## 5. Discussion

In this study, HNT-doped antimicrobials showed different release profiles but all displayed an overall pattern of slow release. Ruiling *et al*. [12] showed that tetracycline hydrochloride-doped HNTs could be electrospun with poly(lactic-co-glycolic acid) to form drug loaded mats that delivered sustained release of its contents, improved the tensile strength of the PLGA fibers, and did not provoke a cytotoxic response [12]. Nitya *et al.* [19] showed similar results using montmorillonite with impressive gains in material properties [19]. We have extended these observations to support the potential applicability of HNTs as a suitable drug-releasing vehicle in a polymer composite. HNTs provided sustained release for a wide array of substances including: anti-bacterial antibiotics, antiseptics, and disinfectants including chlorhexidine, povidone iodine, Brilliant Green, iodine, doxycyclin, and amoxicillin and potassium calvulanate. In addition, amoxicillin, Brilliant Green, chlorhexidine, povidone iodine, and potassium calvulanate were successfully loaded in halloysite-PCL, halloysite-PLA and halloysite-PEO mats. Doped HNT loaded PLA or PEO electrospun scaffolds were also fabricated. However, these mats showed low tensile strength and were rejected for further testing. However, both are resorbable materials and there may be an application using these materials as a drug-releasing platform where tensile strength is not a key consideration.

HNT/PCL mats could be spun in either a random or oriented fiber paper, and fibers also increased in coarseness with HNT content. Drugs and disinfectants could be easily doped into the PCL/HNT fiber mats and released in a sustained fashion. This suggests that halloysite could be used for loading different sets of antibacterials, disinfectants, drugs or active agents and delivered in novel combinations designed to treat specific affected tissues, wound, or diseased organs, or customized to meet a patient’s needs. The amount of drug released from scaffolds is a function of its volume and concentration of HNTs. To have a balance between drug release and material properties, the concentration of HNTs must be limited to 7.5% by weight. Varying the total thickness of the scaffolds can provide with desired drug release volume. This can be achieved in two ways, electrospinning thicker scaffolds or having multiple layers of thinner scaffolds. Scaffolds made through latter method may have better drug release and material properties and also have ease of fabrication. Further investigation on this area is needed to have a definitive proof.

It can be observed that drug release was extended by hours upon loading them in HNTs. Incorporating these doped HNTs into PCL scaffolds has extended this release to days. It is to be noted that this release is from the doped HNTs on the surface of the scaffolds. As the scaffolds are metabolized, more drug release can be expected. Accounting the surface release and further release from matrix, it can be expected that drug release can be extended to weeks or months. Drug release and kinetics from PCL scaffolds are functions of solubility of drug, method of doping HNTs, concentration of doped HNTs in the scaffolds, thickness of scaffold, flux of fluids surrounding the scaffolds, *etc*.

Halloysite nanotubes are currently under active investigation as carrier and container materials for a diverse set of biomedical applications due to their morphology, size, and ability to deliver a diverse set of biologically active agents including antibiotics, disinfectants, anti-cancer agents, growth factors, *etc.* [12,20,21,22,23,24,25,26]. HNTs have been incorporated into various bone cements [7,23], hydrogels [24,25], and polymers [12,13,14,26]. In all cases, HNTs released antibiotics, growth factors or chemotherapeutic agents in a sustained fashion. The desirable cytocompatibility and tunable properties of clay halloysite nanotubes (HNTs) have been established [10,23,24,25,26,27], as well as its potential biocompatibility [10].

A strong driver supporting HNT incorporation into medical biomaterials (e.g., coatings, dressings, implants, *etc.*) is the growing incidence of hospital-acquired infections, the need for novel drug delivery systems, and nanoenhanced scaffolds. A potential HNT application is a flexible and tunable multi-layer wound dressing that possesses multiple functionalities including absorption, antibacterial/fungal protection, and tissue regeneration. The dressing could be used for both prophylactic and therapeutic interventions, as a dressing or wound packing material, or as a topical gauze or pad. The addition of doped HNTs provides enhanced material properties, potential to load multiple drug sets, and increased control over the release kinetics of loaded drugs (10–100+ hrs.) as compared to conventional compositions, properties ideal for the treatment of chronic un-healing wounds, multiple microbial infections, and or for multi-vector treatments.

Halloysite has several advantages over the existing competitor technology, carbon nanotubes. Carbon nanotubes (CNTs) have potential novel application in nanomedicine as biocompatible and supportive substrates, and as pharmaceutical excipients for creating versatile drug delivery systems [28]. Carbon nanostructures are one of the few classes of engineered nanomaterials that have received focused toxicological characterization [29,30]. At present, some emerging concepts of carbon-nanotoxicology can be identified with toxicity dependent on several different factors, such as their size, their shape, the surface characteristics and the amount of the substances present in the particles preparations [23]. In contrast to carbon nanotubes, halloysite nanotubes are significantly cheaper [2,6,7,11], does not provoke a cytotoxic cellular response [2,10,13,23,24,25,26], the inner and outer surfaces are modifiable, and the lumenal diameter fits many globular protein diameters. Furthermore, the large surface area, low cost, ease of loading, tunability and potential broad applicability for medical device development, and no limit on scalability for commercial applications, make HNTs superior to carbon nanotubes.

While we originally targeted HNT/PCL mats for anti-bacterial use, these nanocomposite scaffolds are also promising in several other biomedical application areas. El-Refaie *et al.*, were the first to propose the electrospinning technique as a vehicle as a drug delivery system using spun polylactic acid and poly(ethylene-co-vinylacetate) fibers for the delivery of tetracycline HCl [31]. Ruiling *et al.* [12] for electrospun PLGA/HNTs scaffolds [12], and Patel [13] for PCL/HNTs microfibrous mats, [13] demonstrated the potential of these scaffolds for sustained drug release. As a drug delivery system, drug release can be modified (potentially controlled) by varying production parameters, such as applied voltage, gap distance, flow rate, composition of polymer solution and collector plate design to obtain fibrous scaffolds with a desired diameter, porosity and fiber pattern. In tissue engineering, fibrous scaffolds have a large surface area, possess a rough surface topography, a malleable nature, and the ability to produce fiber architectures patterned after the character of a tissue’s natural extracellular matrix (see Figure 3 and Figure 5). These are design features that support cell adhesion, proliferation, and functionality and would promote neotissue formation [32]. The addition of HNTs to various polymers may enable development of a diverse set of HNT enhanced materials [12,14,19,20,21,22,23,24,25,26,27]. The capabilities of HNTs support their use in constructs that will localize the distribution of chemotherapeutics or biologically active agents at the targeted site and provide sustained drug or agent release, hence increasing drug efficacy and lowering toxicity of the released therapeutics.

## 6. Conclusions

This study employed the use of HNTs as nanocontainers, as well as enhancers of structural integrity, in electrospun PCL scaffolds. HNTs were loaded with amoxicillin, Brilliant Green, chlorhexidine, doxycycline, gentamicin sulfate, iodine, and potassium calvulanate. Selected doped halloysite nanotubes (containing either Brilliant Green, amoxicillin and potassium calvulanate) were mixed with PLC and electrospun into nanocomposite mats. Release profiles from doped HNTs and doped HNT/PCL nanocomposite mats showed a pattern of sustained release for all bacterial agents. Nanocomposites were able to inhibit bacterial growth for up to one-month with only a slight decrease in bacterial growth inhibition. We propose that anti-microbial drug doped HNT/PCL nanocomposites have the potential for use in a variety of medical applications including sutures and surgical dressings. 
